# The Treatment of Empyema

**Published:** 1888-09

**Authors:** Hunter P. Cooper

**Affiliations:** Atlanta, Ga., Professor of Chemistry, Atlanta Medical College


### THE TREATMENT OF EMPYEMA.*

BY HUNTER P. COOPER, M. D., ATLANTA, GA., PROFESSOR OF CHEMISTRY, ATLANTA MEDICAL COLLEGE.

A brief recital of the leading features of the following case, together with a description of the operation performed, will furnish a text on which I will base my remarks:

William Sutherland, aged 23, of Providence, R. I., was admitted to the surgical division of the Presbyterian Hospital during the early part of 1885. For over a year he had been suffering with very severe symptoms, referable to the right side of the thorax, consisting of pain, cough, expectoration, fever, emaciation, and finally a spontaneous discharge of pus from the pleural cavity.

The opening which took place in his chest occurred, without surgical interference, about six or seven months previons to admission. A free discharge of pus from this opening (situated in the second intercostal space in front) continued until his entrance to the hospital. Under the steady drain of this discharge his general condition rapidly depreciated and hectic fever ensued.

When he first entered the hospital, examination of the patient revealed the following condition:

Emaciation and anæma are well marked; pulse and breathing accelerated; in respiration only the left side of the chest expands; on the right side the chest-wall is rigid and sunken; the ribs are crowded together and very tender on pressure. There is an opening in the second intercostal space about three or three and a half inches from the median line. This opening gives vent to a large quantity of creamy yellow pus every time the patient coughs or makes muscular exertion. From the second intercostal space downward there is flatness on percussion, the whole cavity of the chest being evidently full of pus. The lung is crowded into the upper and posterior part of the thoracic cavity,

* Read before the Medical Association of Georgia. From the Transactions.

and judging from the length of time it has been subjected to pressure, it has evidently undergone carnefaction.

Clearly the case was one for surgical interference, and the following operation was performed by Dr. Charles K. Briddon, the attending surgeon:

The patient being etherized, the chest is scrubbed with soap and water, then washed with mercuric bichloride solution (1-1000). A vertical incision was then made in the mid-axillary line, and rapidly deepened until the underlying ribs (5th, 6th, 7th and 8th) were reached. The soft parts were then dissected back until about three and a half inches of each rib were exposed. Portions three and a half inches long of three of these ribs were then excised, the fourth not being removed on account of the patient's feeble condition. To excise the ribs an incision is first made through the enveloping periosteum about four inches long, and the periosteum carefully separated from the bone until a chain-saw can be passed underneath the rib between it and its periosteum. Thus the bone is removed without any danger of wounding the intercostal artery. An incision was made in the 8th intercostal space, giving vent to a large amoumt of pus. The spontaneous opening in the 2d intercostal space was then enlarged, and a large red rubber drainage tube, perforated laterally, was passed from lower to the upper opening, thus securing perfect through-drainage. The chest cavity was then washed out, the wound stitched up, the drainage tube secured in position by safety-pins and adhesive straps, and a very large, thick dressing applied. I should remark that Dr. Briddon carried out antiseptic methods most scrupulously during the entire operation. Patient suffered from moderate degree of shock, but reaction was brought about by the free use of stimulants and hot applications to the surface of the body. For a few days subsequent to the operation, the usual amount of surgical pyrexia was present. The dressing was changed each day, as it became saturated with pus, and the chest cavity washed out, the nozzle of the irrigator being inserted into the drainage tube for this purpose. After a few weeks the discharge had markedly diminished, as had also the capacity of the pus-secreting cavity. The patient's appetite was good; he was free from fever and gaining flesh and strength. He continued to improve steadily until he was discharged in the spring at his own request. At this time his condition was vastly better than it had been at any time, and he was considered on the high road to recovery.

In a letter from Dr. Briddon, dated March 15th, 1886, he says: "It was my desire to make further resection in his (Sutherland's) case. There is still a fistulous communication with a cavity that contains about four ounces of pus, but the man has wonderfully improved."

At the time of the operation the cavity contained nearly a *quart* of pus. It is therefore highly probable that a resection of two or three more ribs would allow sufficient contraction of the chest to close the cavity existing at present.

This case serves to introduce my subject, and is also very instructive, as it shows what may be done for a class of cases formerly hopeless.

The process by which cure is effected in empyema must be considered in order to understand the *rationale* of surgical treatment. In an acute empyema, the pleural surfaces are covered with fibrin and pus, and the cavity contains pus, fibrin and serum in varying proportions. The lung is compressed according to the amount of fluid in the pleural cavity, but its structure has undergone no alteration. Should cure follow the removal of the purulent effusion, there is no obstacle to the expansion of the lung, and the patient recovers without contraction of the chest; and, with the exception possibly of a few adhesions between the lung and the chest-wall, the lung is in as good condition as before the attack. Unfortunately such a result occurs very rarely, only in those few cases where cure has followed aspiration of the chest.

In a chronic empyema there is a very different state of affairs. Here the pleura is very much thickened by a production of new connective tissue, and its surface secretes pus in greater or less quantities. The lung, moreover, having been for a long time subjected to the pressure of the pus, has suffered structural alterations. The prolonged compression has produced collapse of the lung tissue, and forced the lung generally into the upper and back part of the thorax. Here it lies totally unable to take part in respiration, and in time becomes reduced to a solid, cake-like mass, bound down by adhesions and incapable of expansion. Hence when the pus is removed from the chest, the lung is unable to expand and fill the vacancy. The process of cure in such cases is by granulation. The surface of the pleura becomes lined with granulation-tissue. As this becomes organized into connective tissue, contraction takes place, approximating the sides of the abscess cavity. Thus the chest-wall collapses, the diaphragm is drawn upward and the lung drawn downward as much as possible. There is a limit, however, to this contraction, by which the abscess cavity is closed, due to the resistance of the bony chest-wall, on the one hand, and to the inability of the lung to expand, on the other. Hence when nature has done her utmost, there probably still remains a cavity containing from a few ounces to a pint of pus escaping by a fistulous opening. This drain is sufficient to cause amyloid changes in the liver and kidneys and end the patient's life. It is in these cases especially that Estlander's operation holds out a prospect of cure.

I will say nothing regarding the medical treatment of this disease. The importance of keeping the patient's strength and nutrition up to the highest point is fully recognized by every one.

The surgical treatment of empyema is somewhat different in children and in adults, the results being vastly better in the former.

I will now take up *seriatim* the different surgical procedures that can be adopted in a case of empyema:

1. *Aspiration.*—Practically, paracentesis was not resorted to as a means of treating empyema until about thirty years ago. To Dr. Bowditch, of Boston, we are indebted for elaborating this therapeutic resource. Since the invention of the aspirator, the trocar and canula are no longer used in performing this operation. With the aspirator, one of the chief dangers attending the operation (admission of air to the pleural cavity) has been done away with.

Aspiration is the simplest of the three operations that are performed for curing empyema. It is indicated in all except very chronic cases. It is very rarely curative; but as it is almost without danger, and *sometimes* cures, it is well worth a trial before resorting to the more serious operation of opening the chest.

Flint, in his "Clinical Medicine," p. 112, says: "In a very small proportion of cases pus does not re-accumulate, and the aspiration effects a cure." Mr. Godlee, of London, reports *fifty* cases of empyema in the *Lancet* of January 9, 1886. Of these, *six* were cured by aspiration alone—four of the patients being children, the other two adults. He regards aspiration as much more likely to effect a cure in children than in adults.

After the first aspiration it is possible that the pus will not re-accumulate. In the large majority of cases, however, this result unfortunately does not occur, and the operation must be repeated. If the pus re-accumulates rapidly after each aspiration, and contains more and more cellular elements, the operation is doing mischief, and we should temporize no longer, but at once open the chest and secure thorough drainage.

The 8th or 9th intercostal space in a line with the angle of the scapula is the best place to introduce the needle of the aspirator. Whatever space is selected, care should always be taken to insert the needle near the upper border of the *lower* of the two ribs bounding the space; this precaution is necessary in order to avoid wounding the intercostal artery.

If the patient is very sensitive or nervous, it is well to inject cocaine subcutaneously previous to the insertion of the aspirating needle.

Authorities differ as to the amount of pus which should be removed at the first aspiration, some advising a removal of only part of the effusion for fear of inducing hemorrhage or syncope. Others remove all the pus at one sitting. Thus in one of Mr. Godlee's cases, he aspirated 52 oz. and in another 90 oz. A safe rule is to discontinue the aspiration as soon as the patient shows any embarrassment of the respiration, any tendency to cardiac failure, or feels an undue amount of pain.

2. *Opening of Chest and Drainage.*—This operation should be resorted to as soon as it is evident that aspiration is not going to effect a cure, and also in cases in which a fistulous opening already exists. In cases of empyema, complicating chronic pulmonary tuberculosis, it is impossible to lay down any fixed rule regarding opening of the chest. Generally it is unadvisable to perform the operation if the tubercular changes in the lung are far advanced. If, however, the pulmonary involvement is slight, the operation may be performed with much benefit to the patient. Each case must necessarily be judged on its own merits. The operation consists in opening the chest cavity by incision in an intercostal space, exploring the cavity with the finger and with a urethral bougie, and the introduction of a drainage tube. Here as elsewhere a strict adherence to antiseptic methods during the operation and in the subsequent dressings is of paramount importance.

The incision should be made either in the 7th or 8th inter-space with the mid-axillary line as its central point, or a little further back between this point and the angle of the scapula. If the ribs have been crowded very close together, it is best to make the incision upon the 8th or 9th rib, instead of in an inter-space, and to remove an inch or more of the underlying rib. This allows not only better drainage, but a more thorough exploration of the cavity by finger and urethral bougie. Gross speaks of the frequency with which the pus is contained in several cavities. By introducing the finger into the chest, we may be successful in breaking through the adventitious membranes separating different cavities and thus secure drainage of them all. After the pus has been evacuated through the incision, the question of washing out the pleural cavity comes up. This apparently simple and harmless procedure may cost the patient his life. All authors mention cases in which fatal syncope comes on during the injection, though the cause of death is unknown. Fortunately such an unhappy termination is rare, but from its occasional occurrence we draw this lesson—use a *harmless fluid* and inject with *great gentleness.* The fluid may consist of a solution of salt, borax, carbolic acid (1 to 200) or iodine in tepid water which has been previously boiled. The injection should be given with a fountain syringe, or irrigator, elevated but very little above the patient's level. With this amount of care the danger of producing syncope is reduced to a minimum, and the patient runs much less risk of septicæmia than if the cavity is left unwashed.

A large drainage tube (preferably of *red* rubber) should now be introduced through the wound. The tube must go deeply into the cavity, its outer end projecting very little from the wound. It is important to fix it securely in position, for it can be sucked into the chest cavity very easily. The wound is then closed and a thick antiseptic absorbent dressing applied. This will probably be saturated with pus in twenty-four hours ; if so, a new one must be applied, the cavity being washed out as before. The drainage tube is to be gradually shortened as the cavity contracts.

Under this treatment the patient may either recover *completely* or make a partial recovery, there still remaining a fistulous opening leading into a cavity of greater or less extent. In the latter case recovery cannot take place, because contraction has reached its limits, and it is impossible for the cavity to fill up by granulation. It is in such a case that Estlander's operation—resection of portions of several ribs—is applicable, for this allows the chest-wall to sink in and granulation to proceed.

3. *Estlander's Operation.*—This operation was devised and put in practice by Professor Estlander in 1877.

It consists in the resection of portions of several ribs, and is suited to cases of empyema of long standing, in which there is no tubercular complication.

Professor Estlander, in his paper on the subject (Revue Mensuelle de Medicine et de Chirurgie, pp. 157, 885), lays great stress on the importance of the surgeon making himself thoroughly acquainted with the nature of the cavity and the direction in which its greatest extent lies. This knowledge can be attained by exploring the cavity carefully with a urethral sound. Then the operation is so performed that the longest portions of the ribs are removed from that part of the chest-wall which is most distant from the lung. The necessary number of ribs may be exposed, either by turning back a flap of the soft parts, or by a T-shaped incision, or by simply a vertical incision, as in Sutherland's case. Portions of from three to six ribs may be removed so as to make an oval opening into the chest. The method of excising a rib has already been described.

After removal of the ribs, the intercostal arteries are to be secured by strong ligatures, and then the periosteum may be cut away. If the periosteum is left undisturbed, bone is reproduced from it, and the object of the operation may be thus defeated.

After the operation this portion of the chest-wall sinks in and the cavity is filled up, though a second operation is often necessary to complete the process of cure.

In the *British Medical Journal*, January 23, 1886, p. 157, Dr. Maclaren relates the case of a boy eighteen years old, from whom he excised portions of the fifth, sixth, seventh, eighth, ninth and tenth ribs for the cure of an empyema of fourteen months' standing, which had been treated in vain by incision and drainage. The final outcome of the operation was a perfect cure, though it took nearly two years before the cavity closed entirely.

